# Infill Strategy in 3D Printed PLA Carbon Composites: Effect on Tensile Performance

**DOI:** 10.3390/polym14194221

**Published:** 2022-10-08

**Authors:** Sofiane Guessasma, Sofiane Belhabib

**Affiliations:** 1 INRAE, Research Unit BIA UR1268, Rue Geraudiere, F-44316 Nantes, France; 2 Nantes Université, ONIRIS, CNRS, GEPEA, UMR 6144, F-44000 Nantes, France

**Keywords:** filament-based model, PLA–carbon fibre, additive manufacturing, filling pattern, tensile performance, fused filament fabrication

## Abstract

Tuning the infill pattern is one of the key features in additive manufacturing to optimise part weight. In this work, the effect of the infill strategy, including rate and pattern type, is studied on the mechanical performance of polylactic acid (PLA)-carbon composite. In particular, three types of patterns and four filling levels are combined. These combinations are evaluated by tensile loading applied on dogbone specimens. In addition, the underlined deformation mechanisms are further explored using filament-based finite element model. The numerical simulation is built from sliced models and converted into 3D meshes to predict tensile performance. The results show that the infill rate has a nonlinear effect on the density of PLA–carbon composites, and its magnitude depends on the complexity of the generated pattern. In addition, tensile loading is found to activate varied modes of shearing and uniaxial deformations depending on the pattern type. This leads to different profiles and rankings of the tensile performance and allows the infill strategy to significantly affect the part performance, along with its density.

## 1. Introduction

Additive manufacturing (AM) is a genuine process of joining materials layer by layer from a digitalised model [1]. The growing interest on additive manufacturing is justified by numerous advantages, among are which the high level of complexity, the weak dependence to tooling, the local control of the structure, and the customisation of the realisations [2]. According to the review paper by Hasanov et al. [2], AM can be a key technology for transformation of conventional manufacturing, allowing for the production of functionally graded materials, for instance. Several processing routes fall within the definition of AM technology, such as Fused Filament Fabrication (FFF)/Fused Deposition Modelling (FDM) [3], or selective laser sintering (SLS) [4]. The review paper on FDM by Vyavahare et al. [3] shows that this processing route is among the most economical routes for processing polymers. The same author show that process optimization, numerical simulation, and post-production are among the hot topics in FDM. In the case of SLS, El Magri et al. [4] show also that the process parameters such as laser power and hatch orientation play a key role in the improving the mechanical performance of produced parts. One of the major routes of improvement in AM is the development of high-performance feedstock materials such as ceramic-based composites [5] to overcome the loss of mechanical performance generally witnessed after processing. For instance, according to the study by Lizzul et al. [6], the life time of parts manufactured using AM technology differs significantly depending on the orientation of the part. This loss in mechanical performance is mainly due to the discontinuities created during the material laying down, especially for FFF. Indeed, Tao et al. [7] noted that the void structure observed using 3D imaging characterisation could affect the performance of printed parts, such as the interlayer thermal transfer or the mechanical strength. Feedstock composites such as poly(lactic acid)/Ti [8] are an example of newly developed materials that combine strength and biocompatibility. This material has been considered in the study by Lee et al. [8] as a bone substitute to ensure good integration with bone tissue. The authors demonstrated that this material has the ability to improve proliferation and differentiation of pre-osteoblast cells while maintaining a sufficient mechanical stability. Among the material candidates considered as a highly performant feedstock filament in FFF is the carbon fibre reinforced composite. According to the recent review by Valvez et al. [9], the material can be used in lightweight structures while relying on its mechanical strength. In the same review, the literature analysis showed several contributions aiming at finding the right process window to improve the mechanical performance of carbon–PLA printed structures. Among the identified process parameters for FFF, the following are named: printing temperature, feed rate, printing speed, and layer thickness. In addition, some other contributions focused on the material design itself by studying the maximum load rate (up to 27% for some studies) and the quality of the bond between the PLA matrix and carbon fibres. Carbon-based filaments can be thus considered as potential fillers for PLA in FFF and the combination of the two materials has received a great deal of attention [9,10]. Raju et al. [10] showed that optimised FFF settings for nano carbon reinforcement in PLA can be achieved to improve the thickness, printing time, and surface roughness. Aup-Ngoen et al. [11] considered carbon-rich biochar as a feedstock material for FFF and showed a decrease of tensile performance, with loads as small as 0.25%. This was explained by the lack of bond between the matrix and the filler. Heidari-Rarani et al. [12] studied the effect of continuous carbon fibres on tensile and bending behaviour of PLA-based composites for FFF. The authors concluded on the leading role of surface preparation of carbon fibres to achieve an improved mechanical performance compared to pure PLA. Yang et al. [13] considered carbon nanotubes as reinforcement in PLA for loads as large as 6% and reported substantial improvement of tensile strength and moderate increase of flexural strength. Liu et al. [14] compared the ranking of different fillers, among which is the carbon–PLA filament for FFF. The authors showed that carbon–PLA has distinct features such as printability, but low mechanical performance compared to the other filaments. The authors explain this low ranking by a weak interlayer bonding. The literature review by Valvez et al. [9] concludes on the lack of literature on the subject due to the issues related to the development of the material and the complexity to achieve acceptable performance driven by the large number of control parameters for printing and the weak interfacial performance.

In the present study, another aspect of PLA–carbon reinforcement is tackled. This is related to the patterns created during the slicing step to optimise the weight within the printed structure. The examination of the literature on this specific point reveals that it is also subject to significant challenges. For instance, Provaggi et al. [15] developed a finite element framework to study the influence of infill pattern and density on the mechanical performance of various feedstock materials, including polycarbonate, acrylonitrile butadiene styrene, and polylactic acid. The authors showed that honeycomb patterns achieve the best compressive performance. Jin et al. [16] considered another approach of path planning without retraction to achieve optimal filling patterns. Although the authors considered fully dense filling patterns, they reported an improvement in printing process by avoiding filament retraction. Steuben et al. [17] considered different types of infill patterns, including egg crate, random, and Poisson’s based infill to improve the stress and strain within the printed structures. The authors show that depending on the infill pattern, the tensile response can vary significantly with more or less efficient load transfer. This particular aspect is studied in this work for the case of carbon–PLA filaments, where both the infill pattern type and density are combined as input parameters and related to the tensile performance of 3D printed structures.

## 2. Experimental Layout

The filament used as a feedstock material for FFF is a PLA–carbon filament with a diameter of 1.75 mm. The filament is purchased from Protopasta company (Vancouver, WA, USA). It is a PLA matrix reinforced by 10% by weight of milled carbon fibres with a maximum particle size of 0.15 mm. The overall density of the filament is 1.3 g/cm^3^. The recommended settings for printing such a filament are a printing temperature larger than of 205 °C, a bed temperature of 60 °C, and a printing speed between 20 and 40 mm/s. The FFF equipment is a commercial printer under the trade name Anycubic 4Max. The tested specimens have a dogbone-like geometry with typical dimensions: 80 mm × 20 mm × 4 mm, where the width at the gauge area is fixed to 10 mm. The geometry of the specimens is adapted according to the ISO 527-1/-2 standard to perform tensile test. The printing parameters are as follows: nozzle diameter 0.4 mm, layer height 0.2 mm, retraction speed 60 mm/s, retraction distance 45 mm, shell thickness 1.2 mm, bottom/top thickness 0 mm, printing speed 50 mm/s, travel speed 60 mm/s, flow rate 100%, printing temperature 220 °C, bed temperature 60 °C, no support, no platform adhesion raft. All slicing steps are performed using Cura 3.6 from Ultimaker. According to this setup, the top and bottom layers were removed to generate patterns free of skin.

**Figure 1:**
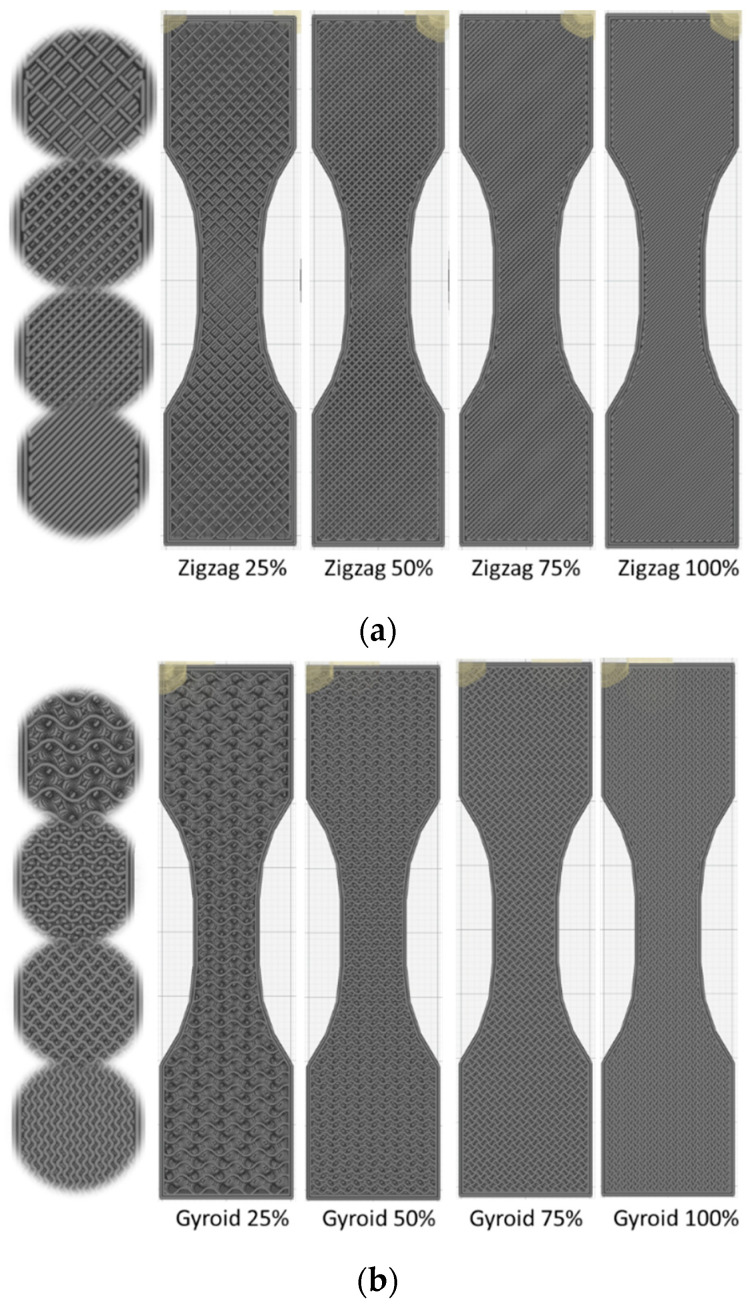

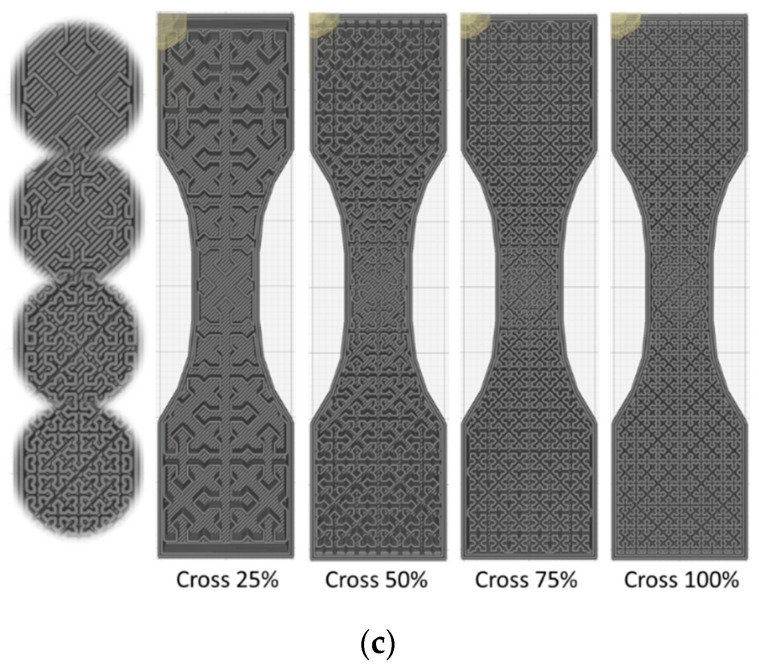
Filling patterns and densities: (**a**) zigzag, (**b**) gyroid, (**c**) cross.

The variables considered in this study are the infill and the pattern type. The infill allows for the adjustment of the density of the realizations. Four levels of infill are selected: 25%, 50%, 75%, and 100%, and three types of patterns are used: cross, gyroid, and zigzag (Figure 1).

All tensile specimens were tested using a Zwick/Roell universal machine equipped with a 10 kN load cell. The tensile loading is performed up to the failure of the material with a displacement rate of 5 mm/min. A total of 52 samples were tested. For each condition (i.e., on average four replicates per condition) the average engineering constants were derived, namely tensile strength, Young’s modulus, and elongation at break. Deformation sequences were monitored using an optical high-speed camera from Photonline (Phantom V7.3). The entire loading sequence is captured in a full frame (800 × 600 pixels) with a moderate speed of 100 fps (frames per seconds).

## 3. Modelling Technique

Finite element modelling is used to explore the deformation mechanisms triggered by the varieties of filling patterns. Structural 3D meshes are generated using cuboid elements starting from the 3D sliced models of the studied patterns. Each element has four nodes and each node is capable of translation in the three main directions (u_x_, u_y_,u_z_). The mesh density is adapted to allow a proper description of the stress and strain fields. For such a purpose, the size of the model is varied between 0.86 × 10^6^ to 2.76 × 10^6^ elements. The material model selected for the PLA–carbon filament is an isotropic linear elastic model with damageable behaviour. Thus, the composite filament is implemented as a homogenised material without considering the detailed microstructural arrangement. In addition, the material is considered to be continuous throughout the thickness, although we know that the building direction is aligned with the thickness of the material. This alignment induces a layering effect that affects the tensile response. However, as discussed later, the effect of the infill in the plane of the construction is more prevailing compared to the out-of-plane behaviour triggered by the layering effect. Both Young’s modulus and Poisson’s coefficients are initially implemented from the datasheet and further adjusted based on the observed tensile behaviour. This adjustment takes into account the filament modification during the laying down process and the possible lack of joining triggered by the layups. The loading conditions are adapted to the case of tensile experiments by constraining the nodes of the bottom and top surfaces against displacement in the loading direction according to (u_x_ = 0 | z = 0; u_x_ = u_0_ > 0 | x = L; x is the loading direction, L is the sample length). The predicted tensile response is built considering the adjustment of all mechanical variables. The stress and strain fields were derived and analysed to derive failure mechanisms. Ansys software was used as a framework for all calculations.

## 4. Results and Discussion

### 4.1. Experimental Evidence

Figure 1 shows the inside view of typical filling patterns used for FFF. By selecting the infill ratio from ratios as small as 25% up to 100%, the rendering of the infill on the mechanical response is completely different depending on the type of pattern. Table 1 shows, for instance, that the density corresponding to the same infill ratio is not the same. For example, cross patterns generate the lowest densities compared to gyroid or zigzag patterns. The analysis of the density data reported in Table 1 shows that a linear correlation exists between the infill ratio and the overall density of the printed carbon–PLA structures. This linear correlation is obtained by running a fitting routine that looks for possible functions with a minimum number of parameters. This correlation can be explicitly related to the pattern type as follows

(1) $$\rho_{G}\left(g/{\mathrm{cm}}^{3}\right)\;=\;0.13\;+\;8.3\;\times \;10^{-3}\;\times \;I_{R}\left(\%\right)\;\;\;|\;R^{2}\;=\;1.00$$

(2) $$\rho_{Z}\left(g/{\mathrm{cm}}^{3}\right)\;=\;0.16\;+\;7.6\;\times \;10^{-3}\;\times \;I_{R}\left(\%\right)\;\;\;|\;R^{2}\;=\;1.00$$

(3) $$\rho_{C}\left(g/{\mathrm{cm}}^{3}\right)\;=\;0.15\;+\;6.8\;\times \;10^{-3}\;\times \;I_{R}\left(\%\right)\;\;\;|\;R^{2}\;=\;1.00$$

where $\rho_{G}$, $\rho_{Z}$, $\rho_{C}$ refer to the overall density of the prints for gyroid, zigzag, and cross pattern filling, respectively, and $I_{R}$ is the infill ratio.

These correlations suggest that the most complex pattern (cross) generates the lowest filling amount. This result supports the idea that an optimal space filling is conditioned by the pattern compactness, which in turn depends on the symmetry of the unit cell composing the pattern.

**Figure 2:**
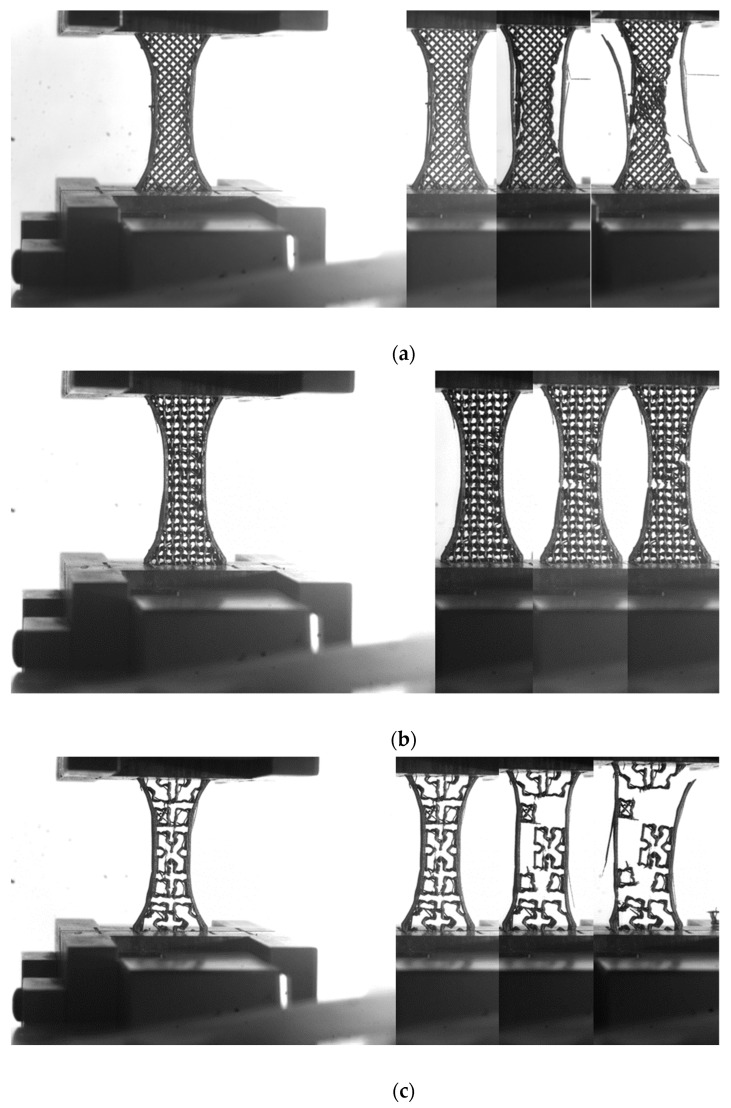
Deformation sequences of airy patterns (infill 25%): (**a**) zigzag, (**b**) gyroid, (**c**) cross.

**Figure 3:**
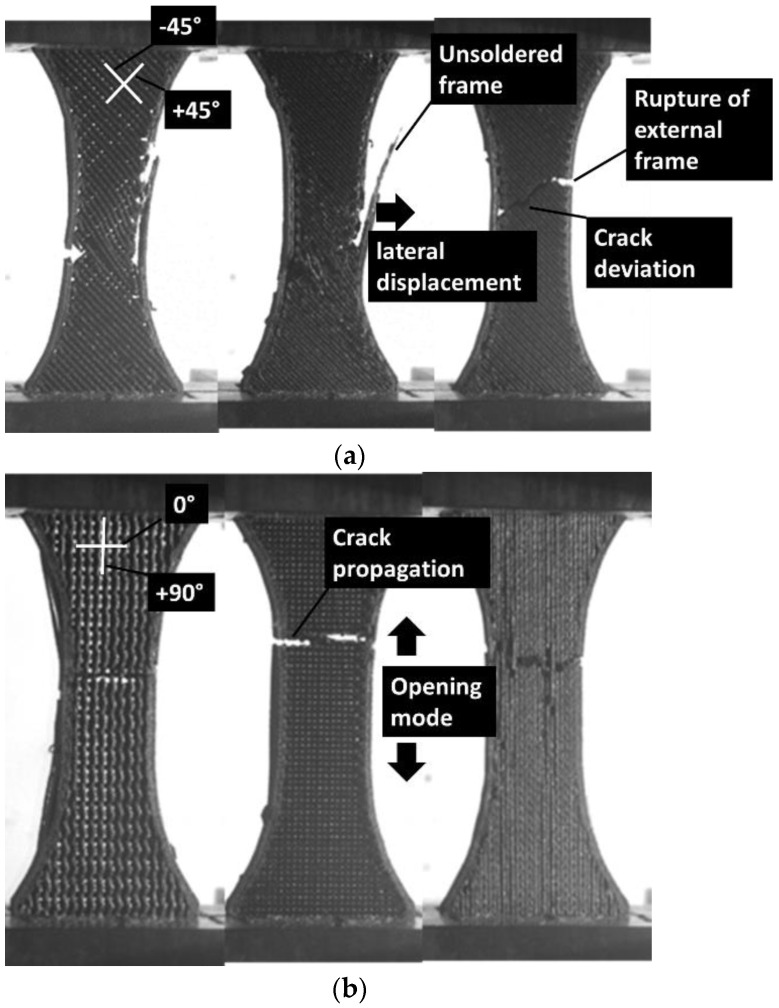

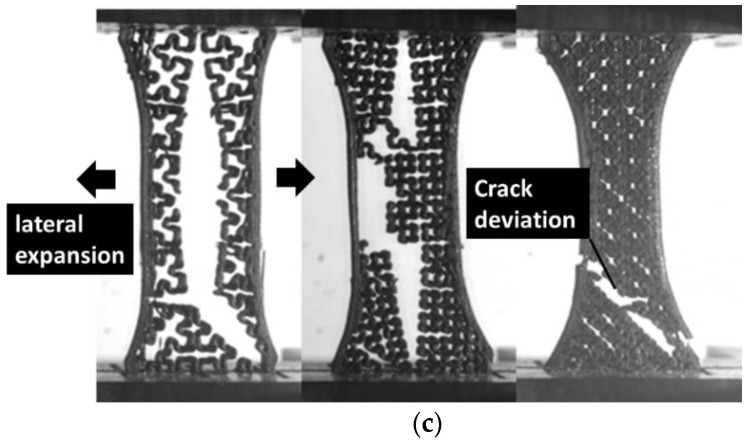
Patterns at the rupture point as a function of density (left to right from 50% to 100%): (**a**) zigzag, (**b**) gyroid, (**c**) cross.

In order to evaluate the effect of such patterns on the tensile performance, deformation sequences of airy and fully dense patterns are examined in Figure 2 and Figure 3.

Figure 2 shows that the tensile loading results in external frame rupture and pattern damage, which is more or less severe depending on the type of the pattern. In the case of zigzag and gyroid patterns, more localised damage is observed. The damage extension is found to be correlated to the direction of filament arrangement within the pattern. This also means that significant shearing behaviour is expected because the cracking initiated within the pattern does not necessarily follow the opening mode (Figure 2a,b). In the case of cross pattern, the damage seems to be to a lesser extent. The tensile loading is marked by more structural displacement within the pattern, and only a few rupture events within the core of the specimen are witnessed (Figure 2c).

Figure 3 shows similar deformation sequences for increasing pattern density. The compactness of the printed PLA–carbon fibre specimens is expected to induce lower deviation from the opening mode, but marked differences are shown. Indeed, as shown in former studies, when the filament stretching capabilities are limited, which is the case for brittle-like filaments (wood–PLA, hemp–PLA, flax–PLA), the deviation from the opening mode is expected to be limited, even if the filament arrangement presents layups of −45°/+45° with respect to the loading direction [18]. For stretchable filaments such as polyamide, strong deviation from the opening mode is observed [19]. In the case of the PLA carbon filament with limited stretching, it would make sense to obtain such a limited deviation. For the zigzag patterns (Figure 3a), the rupture is triggered by stress localisation at the external frame. From this location, cracking is initiated, and the crack propagation follows the pattern. Indeed, the filament arrangement within the pattern was sequenced in a series of +45°/−45° layups. The crack is deviated according to these directions and significant shearing takes place before achieving the rupture point. For a fully dense pattern, the failure of the material is also marked by a significant unsoldering of the external frame from the core of the specimen by a mechanism of Poisson’s expansion. In the case of gyroid pattern (Figure 3b), the filament arrangement highlights two main directions: normal and parallel to the loading direction. This pattern the cracking to be more easily initiated from the external frame and propagated following the opening mode. Only minor deviation from the opening mode is observed. The most contrasted situation is revealed by the cross pattern, which seems to trigger varieties of deformation mechanisms depending on the density (Figure 3b). When the part filling is not fully dense, significant structural displacement and lateral expansion are observed. Damage within the core of the sample seems limited, but with a large extension due to the complexity of the pattern. A fully dense pattern seems to promote instable cracking, for which the propagation is significantly deviated from the transverse direction and correlated to the filament arrangement.

**Figure 4:**
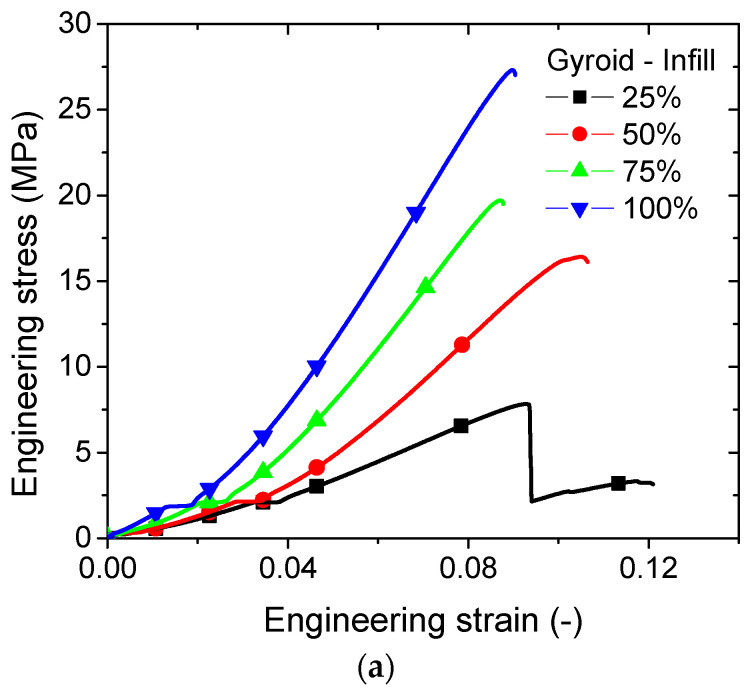

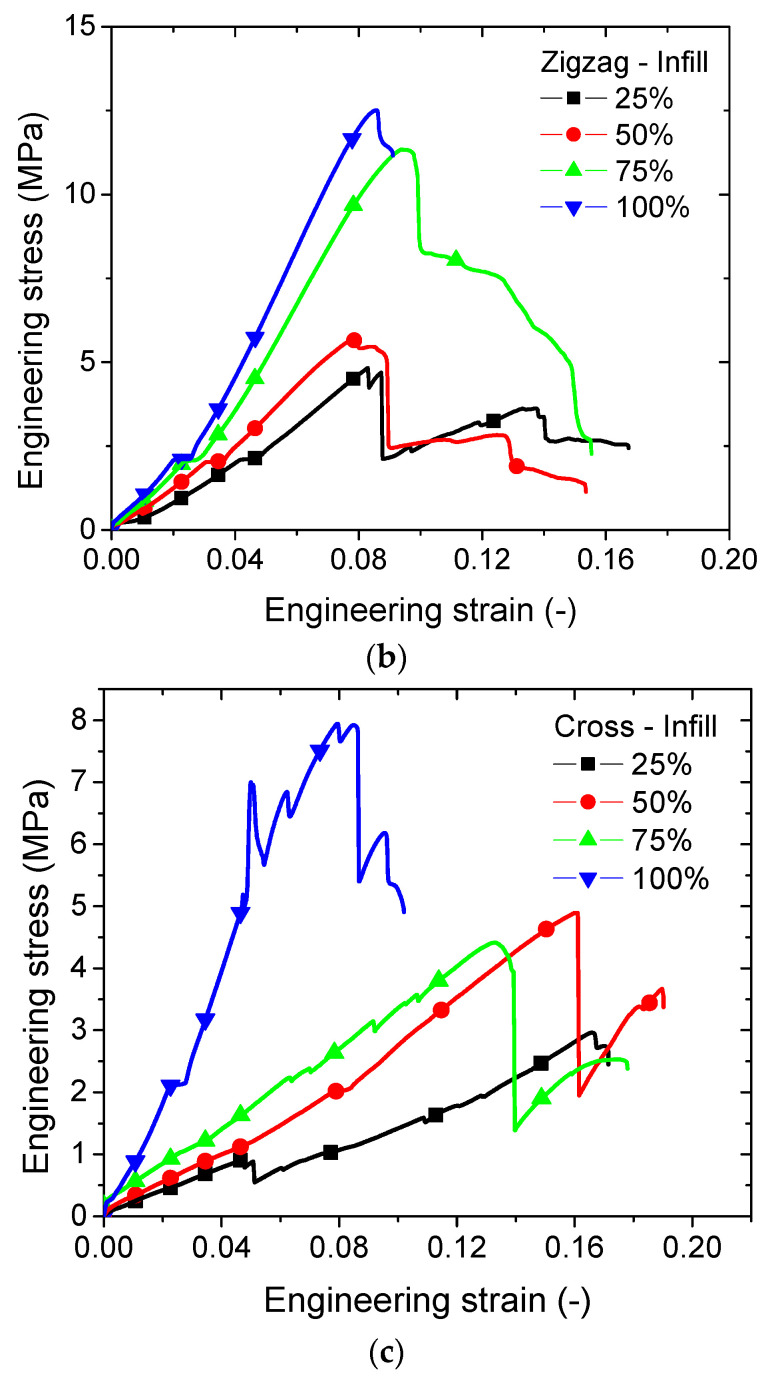
Tensile response as a function of infill and pattern type. (**a**) zigzag, (**b**) gyroid, (**c**) cross.

Figure 4 exhibits the tensile response of all tested PLA–carbon fibre patterns as a function of the infill rate and pattern type. All patterns show brittle behaviour typical of carbon reinforced structures with more or less marked rupture events. The tensile response of a gyroid pattern seems to be smoother with a limited jaggedness, indicating a small number of local rupture events (Figure 4a). The ranking of mechanical response of the gyroid type pattern is the highest among the tested pattern types, irrespective of the infill ratio. The ranking of the tensile response of the zigzag pattern comes in the next position. A larger number of rupture events induces a delayed failure of the material, and the overall response is more complex. In particular, an abrupt rupture event is observed at a loading level of 8%, which corresponds to a critical load transfer to the external frame. The cross pattern is found to generate the lowest tensile response among the studied patterns (Figure 4c). The complexity of the pattern is found to be the cause of the numerous localised rupture events and the resulting jaggedness of the tensile curve.

**Figure 5:**
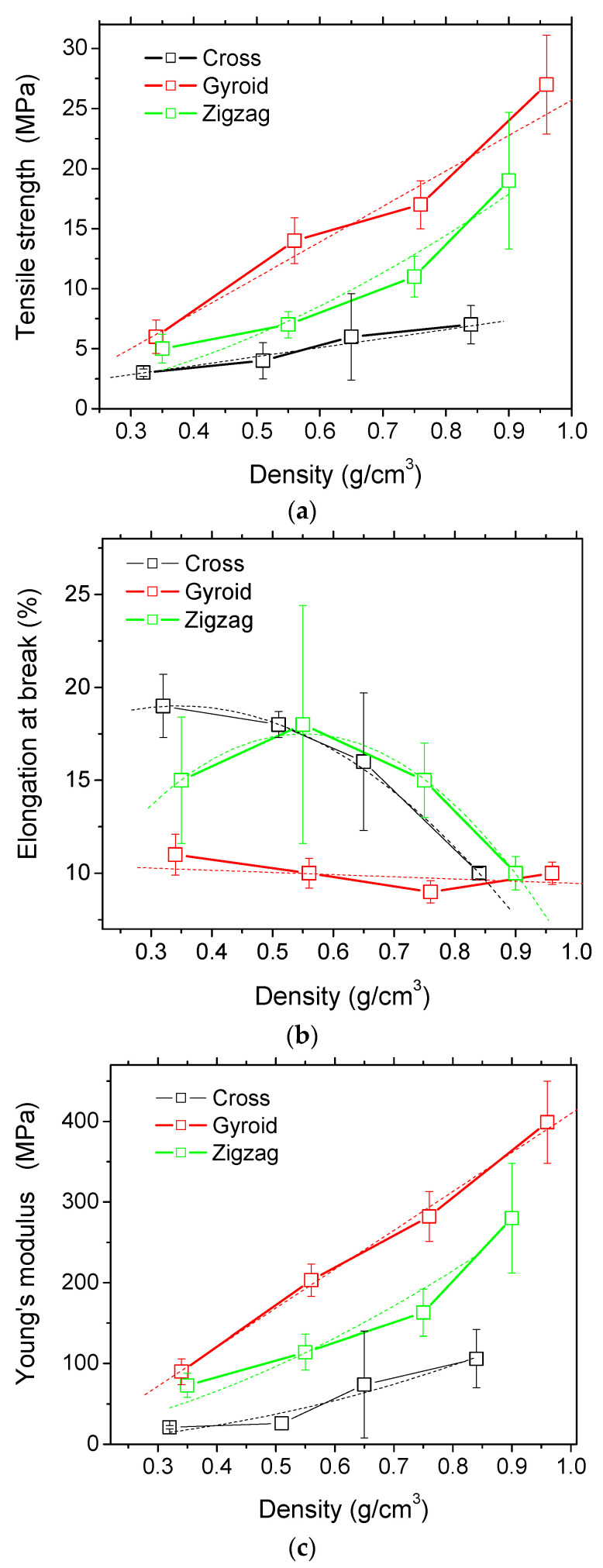
Correlations between engineering constants and the density of PLA–carbon fibre patterns: (**a**) tensile strength, (**b**) elongation at break, (**c**) Young’s modulus.

The correlation between the engineering constants (Young’s modulus, tensile strength, elongation at break) and the infill ratio can be captured from Table 1. The increase of the infill ratio has a direct consequence on the improvement of stiffness and strength and the decrease of elongation at break of the printed PLA–carbon fibres. The ranking of all engineering constants with regards to the type of the pattern is also evidenced. This ranking from the best performing to the lowest is as follows: gyroid, zigzag, cross. According to the data reported in Table 1, the trend exhibited by each pattern is represented by the density–engineering constant curves shown in Figure 5.

From the results shown in Figure 5, the correlation between the overall density of PLA–carbon fibre printed structures and the related engineering constants can be approximated, where appropriate, as linear functions. These correlations highlight a large scatter observed in Figure 5 where, for instance, the error bar for cross patterns (at 0.65 g/cm^3^) is larger compared to the other points. Similar observations can be found in Figure 5b (zigzag) and Figure 5c (cross). The error bars overlapped indicate that the difference is not statistically significant. Figure 5a shows that the tensile strength stretches from low levels of 3 MPa up to 27 MPa. The use of gyroid pattern results in the best tensile strength, followed by zigzag and cross patterns. According to the hypothesis of linear correlations, the following quantified relationships are derived for the tensile strength:

(4) $$\sigma_{C}\left(\mathrm{MPa}\right)\;=\;0.59+7.51\;\times \;\rho_{C}\left(\%\right)\;\;\;|\;\rho_{C}>0\;;\;R^{2}\;=\;0.99$$

(5) $$\sigma_{G}\left(\mathrm{MPa}\right)\;=\;-3.86\;+\;29.57\;\times \;\rho_{G}\left(\%\right)\;\;\;|\;\rho_{G}>0\;;\;R^{2}\;=\;0.96$$

where subscripts *C*, *G*, and *Z* refer to cross, gyroid, and zigzag, and σ, E, and ε refer to tensile strength, Young’s modulus, and elongation at break.

For the zigzag pattern (Figure 5a), the linear correlation has a limited validity (*R*^2^ = 0.88), and the best function that fits the reported tensile strength values is a power law function of the form

(6) $$\sigma_{Z}\left(\mathrm{MPa}\right)\;=\;21.65\;\times \;\rho_{Z}{}^{1.82}\left(\%\right)\;\;\;|\;\rho_{Z}>0\;;\;R^{2}\;=\;0.93$$

The reported data for the elongation at break are more scattered and evolve between 9% and 12%. A distinct feature is the relative weak dependence of the elongation at break on the density for the gyroid pattern compared to the other patterns. The correlations obtained for this engineering parameter, with respect to the density, are nonlinear (Figure 5b).

(7) $$\varepsilon_{C}\left(\%\right)\;=\;14.57\;+\;25.61\;\times \;\rho_{C}\left(\%\right)\;-36.96\;\times \;\rho_{C}²\left(\%\right)|\;\rho_{C}>0\;;\;R^{2}\;=\;1.00$$

(8) $$\varepsilon_{G}\left(\%\right)\;=\;10.63\;-\;1.17\;\times \;\rho_{G}\left(\%\right)\;|\;\rho_{G}\;>\;0\;;\;R^{2}\;<\;0.80$$

(9) $$\varepsilon_{Z}\left(\%\right)\;=\;-1.08\;+\;67.57\;\times \;\rho_{Z}\left(\%\right)\;-\;61.40\;\times \;\rho_{Z}²\left(\%\right)|\;\rho_{Z}\;>\;0\;;\;R^{2}\;=\;1.00$$

Young’s modulus tendencies shown in Figure 5c are similar to the ones depicted for tensile strength and represent the most significant variations (from 21 MPa up to 399 MPa in the full scale). Most of the correlations are captured using the power law function, which is the law known for representing the tensile behaviour of cellular materials.

(10) $$E_{C}\left(\mathrm{MPa}\right)\;=\;155\;\times \;\rho_{C}{}^{2.05}\left(\%\right)\;\;\;|\;\rho_{C}\;>\;0\;;\;R^{2}\;=\;0.94$$

(11) $$E_{G}\left(\mathrm{MPa}\right)\;=\;-73\;+\;482\;\times \;\rho_{G}\left(\%\right)\;\;\;|\;\rho_{G}\;>\;0\;;\;R^{2}\;=\;0.99$$

(12) $$E_{Z}\left(\mathrm{MPa}\right)\;=\;314\;\times \;\rho_{Z}{}^{1.07}\left(\%\right)\;\;\;|\;\rho_{Z}\;>\;0\;;\;R^{2}\;=\;0.93$$

### 4.2. Finite Element Predictions

**Figure 6:**
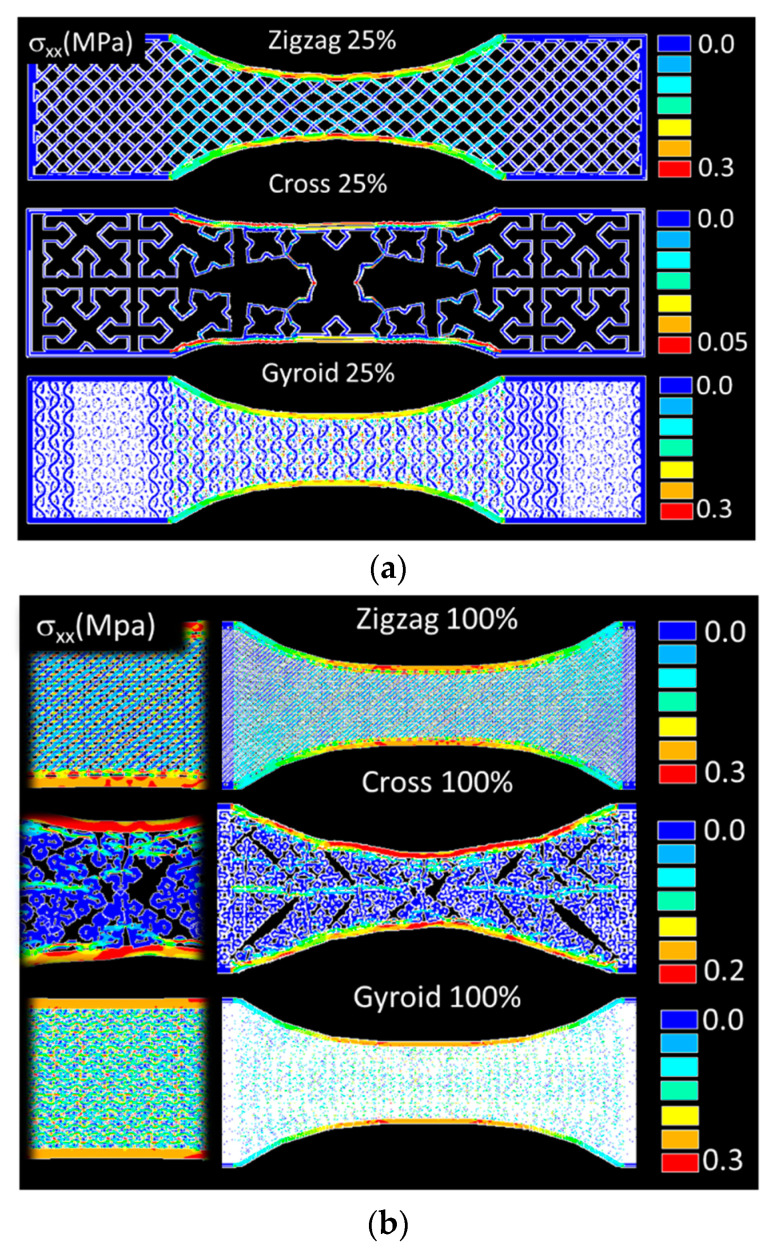
Predicted stress component σ_xx_ field for the three studied filling patterns. Infill ratio (**a**) 25%, (**b**) 100%.

The purpose of the FE computation is to study the deformation mechanisms associated with the observed structural displacements in Figure 2 and Figure 3. This is considered through the predicted stress distributions generated by the infill strategy. Figure 6a shows the predicted distribution of the stress component σ_xx_ of the airy patterns (filling ratio of 25%), where x represents the longitudinal and loading direction. The counterplots confirm a significant load-bearing capability of the external frame, where the largest stress levels are found within this space. Depending on the type of the pattern, the load transfer to the core the specimen varies. For instance, the gyroid pattern is found to promote more load transfer compared to zigzag or cross patterns. Alternation of low and high stress levels is typical in this situation. Stress concentration, in the counterpart, is found within the cross pattern, indicating the preferred sites for local ruptures. The multiplicity of these sites of peak stress levels is in line with the jagged tensile response found for this pattern (Figure 4c). Additionally, the ranking of the studied patterns can be interpreted from the deformed structures. In the case of gyroid, the pattern is a true 3D arrangement of filaments, allowing one of two successive layers to have filament alignment along the longitudinal direction. These filaments exhibit a uniaxial tension along the loading direction and improve the stiffness of the entire 3D structure. The zigzag pattern has no filament aligned with the loading direction but a 45° layup, allowing part of the loading to be transferred as uniaxial tension and the other part as shearing. In the case of cross, the airy structure promotes more filament bending, which induces the localised stress figures shown in Figure 6a. These marked differences in terms of deformation mechanisms establish the nature and ranking of the loading responses depicted in Figure 4.

Figure 6b shows the stress component σ_xx_ counterplots for the same patterns with the maximum filling ratio. All patterns share the same reinforcing effect of the external frame, which is materialised by large stress levels in this part of the specimen. The largest structural displacement depicted in the case of the cross pattern demonstrates that complex patterning triggers low stress levels due to the low load transfer within the core of the specimen. The other patterns alternate low and high stress levels in the core, allowing more load to be supported.

**Figure 7:**
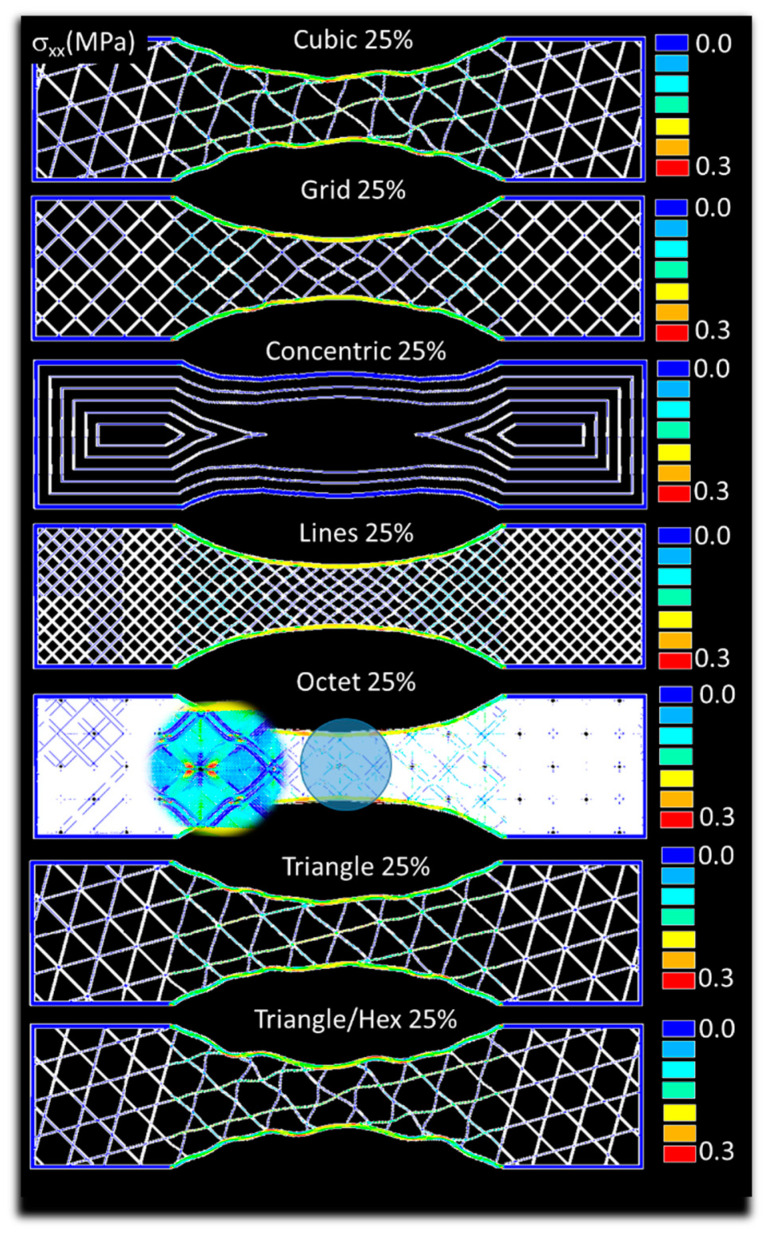
Predicted stress component σ_xx_ field for varieties of airy filling patterns (infill ratio 25%).

**Figure 8:**
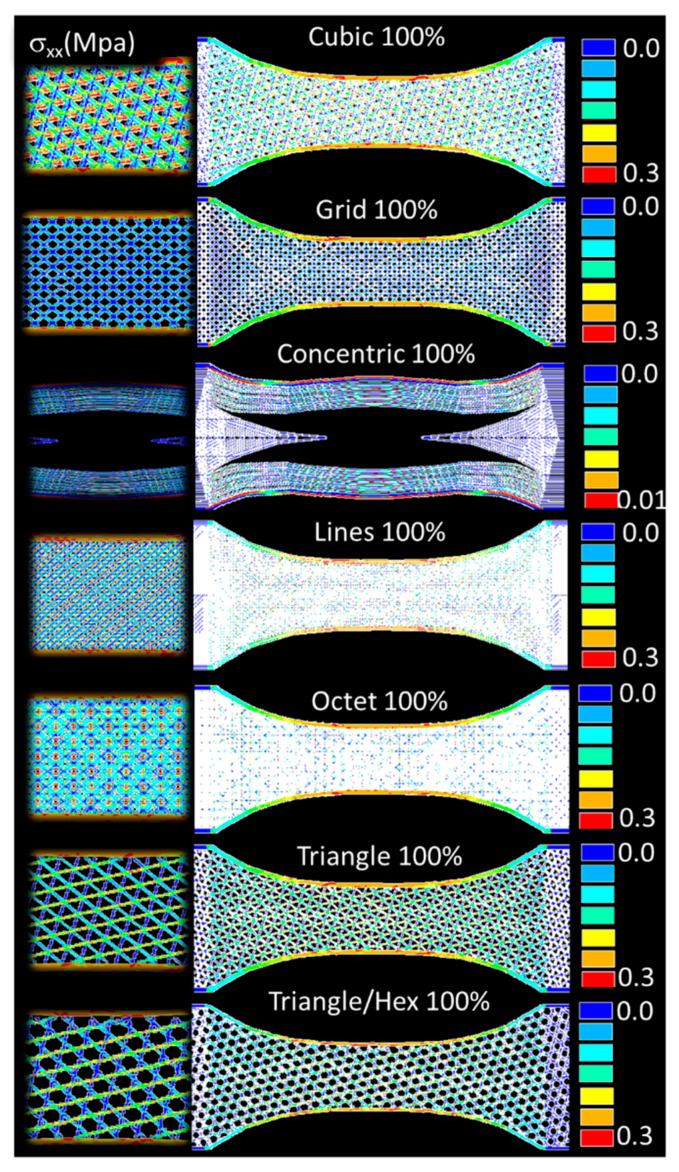
Predicted stress component σ_xx_ field for varieties of airy filling patterns (infill ratio 100%).

Figure 7 exhibits the predicted stress counterplots for the case of airy cubic, grid, concentric, octet, triangle/hex like patterns. All these patterns correspond to an infill of 25%. Significant variations in the stress fields are observed depending on the density of the pattern. For instance, triangle and triangle/hex like patterns induce bending at the external frame, while strong shearing is predicted for the case of octet pattern. In addition, low stress transfer is found in the case of concentric pattern because of the lack of pattern connectivity. It has to be mentioned that the balance between the load transfer between the external frame and the raster also varies significantly depending on the pattern type. The frame is found to maintain a strong contribution to the load transfer for most of the patterns with different magnitudes, except for the case of concentric pattern. Figure 8 shows the predicted stress fields for the same patterns, but with 100% of infill rate. These structures exhibit higher structural stability, but the same stress heterogeneity develops due to the porosity generated by the material discontinuity. The tensile loading generates a mix of deformation mechanisms that encompass uniaxial deformation and shearing. However, external frame bending vanishes as the infill rate increases. This is, for instance, the case for the triangle and triangle/hex like pattern. It has to be mentioned that even with a higher density achieved with the 100% infill, negative stress values are also predicted within the raster due to the nature of the load transfer. This is the case, for instance, for the octet pattern. Even if this is minor, compressive stress field also indicates the complexity of the deformation mechanisms induced by adopting filling patterns.

## 5. Conclusions

This study concludes that the infill strategy affects the density and the mechanical performance of the printed material differently. For same infill rate, gyroid, zigzag, or cross infill do not lead to the same density nor the same tensile performance. The type of the pattern is found to influence intensity and the balance between the deformation mechanisms, including tension, shearing, and bending. The difference between load transfer to the external frame and the raster is also a leverage that the type of infill is capable of modulating. This difference is found to be dictated by the nonlinear correlation between the infill rate and the density, as well as the generated pattern connectivity. Gyroid pattern is found to be the best option to improve the mechanical strength, while zigzag and cross are found to be more suitable for promoting large stretching, especially at low infill rates.

## Figures and Tables

**Table 1:** Density and engineering constants of carbon–PLA patterns.

Pattern	Infill (%)	Density (g/cm^3^)	Tensile Strength (MPa)	Elongation at Break (%)	Young’s Modulus (MPa)
Gyroid	25	0.34 ± 0.03	6 ± 1.4	11 ± 1.1	90 ± 16
	50	0.56 ± 0.04	14 ± 1.9	10 ± 0.8	203 ± 20
	75	0.76 ± 0.06	17 ± 2.0	9 ± 0.6	282 ± 31
	100	0.96 ± 0.07	27 ± 4.1	10 ± 0.6	399 ± 51
Zigzag	25	0.35 ± 0.03	5 ± 1.2	15 ± 3.4	73 ± 15
	50	0.55 ± 0.05	7 ± 1.1	18 ± 6.4	114 ± 22
	75	0.75 ± 0.07	11 ± 1.7	15 ± 2.0	163 ± 29
	100	0.90 ± 0.09	19 ± 0.3	10 ± 0.9	280 ± 68
Cross	25	0.32 ± 0.04	3 ± 0.5	25 ± 1.7	21 ± 2
	50	0.51 ± 0.05	4 ± 1.5	18 ± 0.7	26 ± 8
	75	0.65 ± 0.06	6 ± 3.6	16 ± 3.7	74 ± 66
	100	0.84 ± 0.07	7 ± 1.6	10 ± 0.3	106 ± 36

## Data Availability

The data presented in this study are available on request from the corresponding author.
